# Treatment of subtrochantric femur fracture with intra-medullary nailing (IMN) in a patient with osteopetrosis: A case report and literature review

**DOI:** 10.1016/j.ijscr.2025.111592

**Published:** 2025-06-30

**Authors:** Ajlan alajlan, Abdulaziz Alkhudhayri, Salem Alshehri, Mohamed almadah, Mohamed Othman

**Affiliations:** a Prince sultan military medical city, Riyadh, Saudi Arabia; b College of medicine, Qassim university, Qassim, Saudi Arabia; c College of medicine, King Saud university, Riyadh, Saudi arabia; d King Fahd Specialist Hospital in Buraydah, Saudi arabia

**Keywords:** Osteopetrosis, Subtrochanteric femoral fracture, Intramedullary nailing, Adult-onset autosomal dominant, Bone density and remodeling

## Abstract

**Introduction and importance:**

Osteopetrosis is a rare hereditary bone disorder characterized by defective osteoclastic bone resorption, leading to dense but brittle bones. These structural abnormalities predispose patients to fractures and complicate orthopedic surgeries due to obliterated medullary canals and increased cortical density. Subtrochanteric femoral fractures in such patients are rare and challenging, with limited consensus on optimal surgical management.

**Case presentation:**

We present a 45-year-old male with adult-onset autosomal dominant osteopetrosis who sustained bilateral subtrochanteric femoral fractures—first on the right side, then on the left two years later. Both fractures were managed with intramedullary nailing (IMN). The procedures were technically demanding due to the sclerotic bone and canal obliteration, requiring sequential drilling and reaming with irrigation to prevent thermal necrosis. Both fractures healed without complications, with radiographic union at 9 months and early mobilization.

**Clinical discussion:**

IMN in osteopetrotic bone is technically difficult but feasible with meticulous planning and technique. Reported complications include drill bit breakage, thermal injury, delayed union, and hardware failure. Compared to extramedullary fixation, IMN offers better mechanical stability in weight-bearing regions. Our experience aligns with other reports suggesting IMN is a viable approach when performed with necessary precautions. The lack of standardized protocols necessitates individualized planning based on anatomical and clinical considerations.

**Conclusion:**

This case highlights the successful application of IMN for subtrochanteric fractures in adult osteopetrosis, emphasizing the importance of surgical expertise. Further studies are needed to guide standardized management in this rare condition.

## Highlights

• Osteopetrosis is a rare genetic condition characterized by brittle, sclerotic bones due to defective osteoclastic activity.
• We report a rare case of bilateral subtrochanteric femoral fractures in a 45-year-old male with adult-onset osteopetrosis.
• Both fractures were managed successfully using intramedullary nailing despite significant technical challenges.
• The case emphasizes the biomechanical and surgical difficulties in treating fractures in osteopetrotic bones.
• Intramedullary nailing was associated with good functional outcomes and bony union without complications.

## 1 Introduction

Osteopetrosis is a rare genetic disorder, marked by defective osteoclast function, leading to dense but fragile bones [1]. It exists in two main forms: a severe, autosomal recessive type (ARO) seen in infancy with high mortality, and a milder, adult-onset autosomal dominant type (ADO) [1]. Fractures, especially in the femur and tibia, are common and heal poorly due to impaired bone remodeling [2]. Historically managed conservatively, treatment has evolved with surgical techniques now enabling successful fixation [3]. This case discusses an adult with ADO who sustained bilateral subtrochanteric femoral fractures, both successfully managed with intramedullary nailing. This case report has been reported in line with the SCARE checklist [4].

## 2 Case presentation

We present the case of a 45-year-old male in line with the CARE criteria [5], with a known diagnosis of osteopetrosis who sustained two separate subtrochanteric femoral fractures, occurring two years apart. The patient initially sustained a subtrochanteric fracture of the right femur, managed with intramedullary nailing. Two years later, a contralateral (left) subtrochanteric fracture occurred, which was also treated surgically with intramedullary nail.

**Fig. 1:**
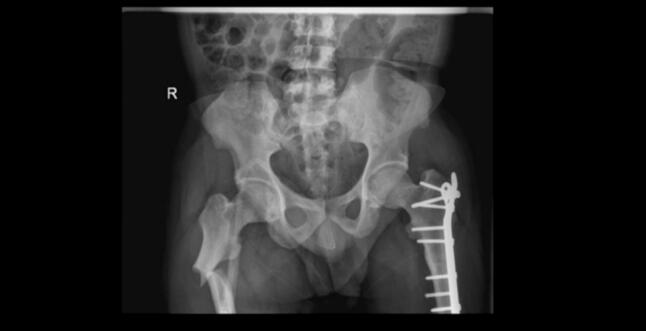
Initial AP image showing the right subtrochanteric femur fracture.

**Fig. 2:**
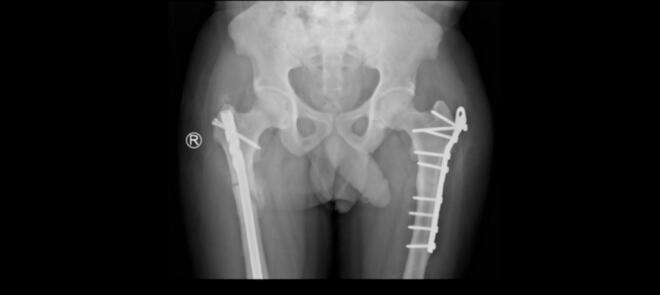
Follow-up image showing healed right femur fracture with callus formation and intramedullary nail.

**Fig. 3:**
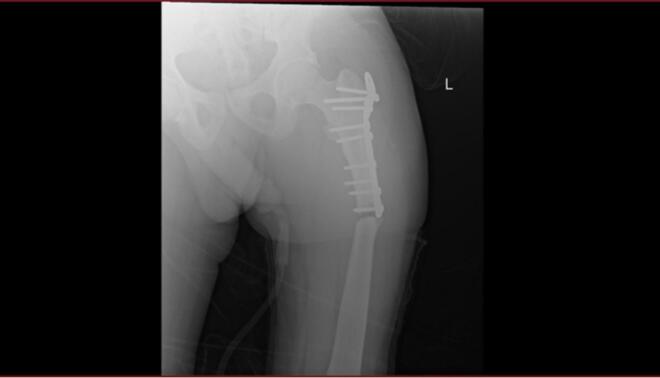
AP radiograph of the left femur showing the previous plate and screws with subtrochanteric fracture.

**Fig. 4:**
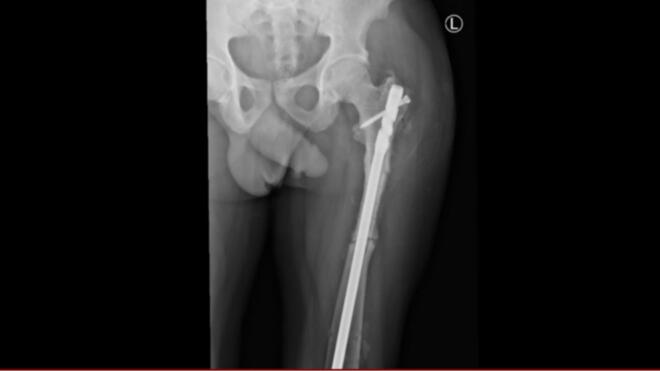
Postoperative AP image showing left subtrochanteric intramedullary nailing with callus formation.

Intraoperatively The patient was positioned laterally, and a lateral femoral approach was utilized. The previous plate and screws were removed for the left subtrochanteric fracture. Upon identifying the fracture site, the medullary cavity was found to be totally consolidated. Canal preparation began with 4.5 mm and then 6.5 mm drill bits, performed retrograde for the proximal fracture fragment and antegrade for the distal fragment. An awl was used to access the greater trochanteric entry point. Flexible reaming started at 9 mm and was escalated to 11 mm to accommodate a 10 mm nail. The reamer was advanced slowly with frequent saline irrigation. An antegrade TAN nail was then inserted and locked proximally and distally. A drain was placed, and the skin was closed in layers. For Operative Duration, the procedure lasted approximately 3.5 h on the left side and 2 h on the right side. Estimated Intraoperative Blood Loss: 450 mL on the left side and 300 mL on the right side, as measured using the suction canister method. Intraoperative challenges included difficulty identifying the medullary canal and resistance to reaming, requiring slow advancement and frequent saline irrigation. A blocking screw was used to correct anterior drift of the nail. Postoperatively, the patient mobilized early and was discharged on the third day. Radiographs showed callus formation at three months and complete union at nine months (Fig. 1, Fig. 2, Fig. 3, Fig. 4).

## 3 Discussion

Osteopetrosis is a rare hereditary disease characterized by an excessive increase in bone density stemming from impaired osteoclastic bone resorption, which results in the accumulation of structurally compromised bone prone to fracture, in severe cases the medullary cavity is extensively occupied by endochondral bone, leaving minimal residual space and thereby significantly contributing to the fragility noted in osteopetrotic bone [6].

A variety of surgical strategies have been documented for the treatment of peritrochanteric fractures in patients with osteopetrosis. However, several complications—including nonunion, implant failure, refracture, and deep infection—have been reported. Specifically, in 25 published cases (4 femoral neck fractures and 21 peritrochanteric fractures) managed using open operative techniques, there was a 12 % rate of nonunion and a 12 % rate of infection. In the peritrochanteric subgroup, hardware failure occurred in 29 % of patients, with a reoperation rate of 29 % and a periprosthetic fracture rate of 14 % when utilizing Hansen-Street nails, plates and screws, Jewett plates, blade plates, dynamic hip screws, and intramedullary nails (IMN). Despite the availability of multiple fixation devices, the most suitable implant for subtrochanteric fractures in osteopetrosis remains under debate [[7], [8], [9], [10], [11], [12], [13], [14], [15]].

Selecting an appropriate surgical method is paramount to reducing fixation failures and ensuring successful completion of the procedure. In the general population, intramedullary fixation is often preferred for proximal femoral fractures because of its superior biomechanical performance under axial loading. Nevertheless, significant long-bone deformities and associated medullary canal abnormalities typically contraindicate IMN [16]. Additionally, the intraoperative challenges associated with locating the medullary canal, difficult reaming, and heat generation during reaming further complicate its use. Notably, IMN with or without osteotomy is sometimes the only reliable choice for certain hereditary bone disorders, including osteogenesis imperfecta and X-linked hypophosphatemic rickets [17].

For femoral fractures associated with osteopetrosis, plate-and-screw fixation is commonly employed, particularly for femoral fractures [18]. The primary challenge in osteopetrotic bone lies in drilling and reaming the exceedingly dense cortex. On the other hand, extramedullary implants carry an elevated risk of soft tissue irritation and fixation failure in subtrochanteric regions compared with IMN [19]. Moreover, extramedullary implants may require prolonged retention due to the defective osteoclast function and impaired bone remodeling characteristics of osteopetrosis.

Kleinberg documented a case of peri-trochanteric fracture treated via plate, screw, and cortical strut allograft. Although the plate ultimately failed and the fracture site became angulated, union was successfully achieved [20]. Similarly, Chhabra described six patients with peritrochanteric fractures: two managed with dynamic hip screws, three with Kuntscher nails (K-nails), and one with a locked intramedullary nail. Both DHSs failed—one secondary to infection and resultant nonunion, and the other due to implant pull-out causing nonunion. While the three fractures treated with K-nails demonstrated eventual union, one nail migrated and necessitated exchange nailing prior to healing. The locked nail case healed without complications [21].

Kumbaraci reported on a patient with bilateral subtrochanteric femur fractures treated using bilateral Proximal Femur Nail Antirotation (PFNA) implants. Intraoperatively, creating an intramedullary canal in the osteopetrotic bone proved challenging, with one drill bit fracturing during the procedure; however, no femoral shaft fractures occurred. Postoperatively, the patient experienced bilateral hip pain but no major complications, and both fractures ultimately achieved union by 12 months [22]. In contrast, we did not encounter similar drill bit failure in our case. This may be attributed to the meticulous technique employed during reaming, along with the selection of an appropriately robust drill bit suitable for navigating the sclerotic medullary canal typically observed in patients with osteopetrosis.

In another report, a patient with a transverse subtrochanteric fracture of the right femur underwent fixation using a 10-hole titanium distal femoral locking compression plate (DF-LCP; Synthes). Intraoperative drilling was challenging due to the dense osteopetrotic cortex; however, no drill bits fractured, and stable fixation was achieved with multiple locking screws. Radiographic union was confirmed by the 23rd postoperative week, after which the patient was permitted full weight-bearing. An incomplete stress fracture of the contralateral femur was identified postoperatively and managed conservatively [23]. In our case, the patient was also able to mobilize early; however, radiographic union was delayed, with soft callus formation first observed at three months and complete union achieved by nine months. This prolonged healing may be attributed to the more complex fracture pattern in our case, which presented with poor initial radiological features, including angulation and deviation of the fracture.

Summary of case reports highlights both the challenges and successful outcomes associated with various fixation methods for subtrochanteric femoral fractures in patients with osteopetrosis.”

**Table:** 

Case	Age/sex	Inheritance type	Fracture features	Management	Complications	Outcome	Citation
1	20/F	Autosomal Dominant Osteopetrosis	Right subtrochanteric femoral fracture with comminution and cortical thickening; radiographs showed generalized osteosclerosis	Open reduction and internal fixation with dynamic hip screw	Intraoperative femoral neck fracture	Complete healing at 12 months and 2 years postoperatively	[24]
2	27/M	Not specified	Bilateral subtrochanteric femur fractures; right side displaced transverse fracture, left side stress fracture; increased bone density on radiographs	Internal fixation and recombinant human bone morphogenetic protein-7	Delayed union on right side; heterotopic bone formation	Both fractures healed; uncertain contribution of bone morphogenetic protein	[25]
3	26/M	Autosomal dominant type II osteopetrosis	Left subtrochanteric fracture with transverse pattern; sclerotic cortices and narrow medullary canal	Intramedullary nailing	Delayed union; gap at fracture site	Fracture healed at 10 months after dynamization	[26]
4	70/F	Autosomal dominant type II osteopetrosis	Bilateral subtrochanteric fractures; transverse pattern with cortical thickening; right femur showed angular deformity due to delayed union.	Plate fixation on right; prophylactic nailing on left	Delayed union on right side	Delayed union on right; healed on left at 8 months	[26]
5	36/F	Type 2 adult osteopetrosis	Right subtrochanteric fracture with oblique fracture line and cortical thickening; radiographs showed generalized osteosclerosis	Intramedullary interlocking nailing	None.	Successful management; technical difficulties noted	[27]
6	52/M	Intermediate autosomal recessive osteopetrosis	Subtrochanteric femur fracture with oblique fracture line and diffuse cortical thickening; no visible intramedullary canal	Dynamic hip screw internal fixation	None	Stable internal fixation; no activity limitations at 15 months follow-up	[28]
7	56/M	Autosomal dominant osteopetrosis	Left subtrochanteric fracture extending into the femoral neck with transverse pattern; ipsilateral comminuted femoral neck fracture	Nonoperative treatment with hip spica cast	Coxa vara and external rotation deformities	Fracture united with deformities; patient returned to normal activities	[29]
8	25/F	Not specified	Transverse subtrochanteric fracture of the right femur with cortical thickening and no visible medullary canal	Not specified	Not specified	Not specified	[30]
9	33/M	Not specified	Bilateral diaphyseal femoral fractures with oblique and transverse patterns; radiographs showed generalized sclerosis	Not specified	Not specified	Not specified	[31]
10	25/M	Not specified	Bilateral subtrochanteric femur fractures with comminuted pattern and cortical sclerosis; no visible medullary canal	Proximal femoral nail antirotation	None	Successful management; technical difficulties noted	[32]

## 4 Conclusion

This case demonstrates the successful management of bilateral subtrochanteric femoral fractures in an adult with autosomal dominant osteopetrosis (ADO) using intramedullary nailing (IMN). Despite the inherent challenges posed by osteopetrosis—including a sclerotic, obstructed medullary canal and increased risk of hardware failure—IMN achieved stable fixation, early mobilization, and eventual bony union. Key technical considerations, such as gradual canal preparation with sequential drilling and reaming, saline irrigation to prevent thermal necrosis, and the use of blocking screws to correct nail drift, were critical to overcoming the anatomical constraints of osteopetrotic bone. The patient's ability to bear full weight post-operatively and achieve union within 9 months underscores the biomechanical advantages of IMN over plate fixation in this high-stress fracture region. However, the prolonged healing time highlights the impaired remodeling capacity of osteopetrotic bone, necessitating extended follow-up. This case aligns with recent literature advocating for IMN as a first-line surgical option for femoral fractures in ADO, provided surgeons anticipate technical difficulties (e.g., instrument breakage, need for specialized tools) and prioritize meticulous technique. Future directions include exploring adjunct therapies (e.g., teriparatide to stimulate osteoclast activity) and refined reaming protocols to further reduce complications. Collaborative, multicenter studies are needed to establish standardized guidelines for fracture management in this rare population.

## CRediT authorship contribution statement

All authors contributed to the patient's care, data collection, and manuscript preparation.

## Consent

Written informed consent was obtained from the patient for publication of this case report and accompanying images.

## Ethical approval

Not required for a single case report, as per institutional policy.

## Guarantor

Dr. Abdulaziz Alkhudhayri.

## Funding

None.

## Declaration of competing interest

The authors declare no conflicts of interest.
